# Mitochondrial HTRA2 Plays a Positive, Protective Role in *Dictyostelium discoideum* but Is Cytotoxic When Overexpressed

**DOI:** 10.3390/genes9070355

**Published:** 2018-07-16

**Authors:** Suwei Chen, Oana Sanislav, Sarah J. Annesley, Paul R. Fisher

**Affiliations:** 1 Discipline of Microbiology, Department of Physiology Anatomy and Microbiology, La Trobe University, VIC 3086, Australia; chensuwei@aku.edu.cn (S.C.); O.Sanislav@latrobe.edu.au (O.S.); S.Annesley@latrobe.edu.au (S.J.A.); 2 School of Modern Agriculture and Biological Science and Technology, Ankang University, Shaanxi 725000, China

**Keywords:** HTRA2, Parkinson’s disease, *Dictyostelium*, endocytosis, growth, mitochondria, phototaxis

## Abstract

HTRA2 is a mitochondrial protein, mutations in which are associated with autosomal dominant late-onset Parkinson’s disease (PD). The mechanisms by which HTRA2 mutations result in PD are poorly understood. HTRA2 is proposed to play a proteolytic role in protein quality control and homeostasis in the mitochondrial intermembrane space. Its loss has been reported to result in accumulation of unfolded and misfolded proteins. However, in at least one case, PD-associated HTRA2 mutation can cause its hyperphosphorylation, possibly resulting in protease hyperactivity. The consequences of overactive mitochondrial HTRA2 are not clear. *Dictyostelium discoideum* provides a well-established model for studying mitochondrial dysfunction, such as has been implicated in the pathology of PD. We identified a single homologue of human HTRA2 encoded in the *Dictyostelium discoideum* genome and showed that it is localized to the mitochondria where it plays a cytoprotective role. Knockdown of HTRA2 expression caused defective morphogenesis in the multicellular phases of the *Dictyostelium* life cycle. In vegetative cells, it did not impair mitochondrial respiration but nonetheless caused slow growth (particularly when the cells were utilizing a bacterial food source), unaccompanied by significant defects in the requisite endocytic pathways. Despite its protective roles, we could not ectopically overexpress wild type HTRA2, suggesting that mitochondrial HTRA2 hyperactivity is lethal. This toxicity was abolished by replacing the essential catalytic serine S300 with alanine to ablate serine protease activity. Overexpression of protease-dead HTRA2 phenocopied the effects of knockdown, suggesting that the mutant protein competitively inhibits interactions between wild type HTRA2 and its binding partners. Our results show that cytopathological dysfunction can be caused either by too little or too much HTRA2 activity in the mitochondria and suggest that either could be a cause of PD.

## 1. Introduction

Mammalian HTRA2 is a nuclear-encoded mitochondrial serine protease which is expressed as a 49 kDa precursor protein that is cleaved to a 38 kDa mature protein upon import into the mitochondria [1]. It includes a mitochondrial targeting sequence, N-terminal transmembrane domain, a serine protease domain and a C-terminal PDZ protein interaction domain [2], named after the first three proteins found to contain this domain: Post synaptic density protein (PSD95), *Drosophila* discs large 1 tumor suppressor (dlg1), and Zonula occludens-1 protein (ZO-1). Under normal conditions, HTRA2 is localized to the mitochondria, but is released to the cytosol after apoptotic stimuli such as UV irradiation and oxidative stress. In the cytosol, it initiates caspase-dependent programmed cell death as well as cell death by caspase-independent (proteolysis) pathways [1,3,4,5].

Mutations in either the serine protease or PDZ domains are reported to be associated with autosomal dominant Parkinson’s disease (PD) [6,7,8]. HTRA2 was also found to colocalize with another PD-linked protein, α-synuclein, in Lewy Bodies, neuronal protein aggregates that are the hallmark of most forms of PD [9]. The role of HTRA2 in PD pathogenesis is thought to result from loss of its ability to remove misfolded or damaged mitochondrial proteins through its serine protease function. Mutations to this function result in the accumulation and aggregation of proteins, which is characteristic of PD. HTRA2 also interacts with other PD proteins such as PINK1, which has been reported to phosphorylate HTRA2 and this phosphorylation at S142 increases its proteolytic activity [10,11,12,13,14]. Amino acid substitutions (A141S, G399S) close to this and another phosphorylation site (S400) have been implicated in PD and are believed to act by inhibiting phosphorylation and enzyme activity [13].

Conflicting data about the role of HTRA2 in PD has also been reported [2]. For example, Simon-Sanchez and Singleton [15] found previously reported pathogenic HTRA2 mutations in neurologically normal controls, while others found no strong association between HTRA2 variants and PD in population genetic studies [16,17,18]. The discrepancies between the negative population studies and the positive studies on PD kindreds carrying HTRA2 mutations may arise from combinations of low penetrance, allele rarity and genetic modifiers.

Regardless of its place in the pantheon of PD-associated proteins, HTRA2 is a mitochondrial protein whose genetic loss of function thus represents a mitochondrial disease. However, there has also been disagreement about the cytopathological effects of HTRA2 loss on the mitochondria, particularly in relation to its role in the Parkin-PINK1 pathway, which affects mitochondrial integrity and dynamics [2,19]. In animals and humans, the complexities of mitochondrial biology are such that the genotype-phenotype nexus is broken—the same mitochondrial defects can produce dramatically different outcomes in different individuals, while different mutations affecting different genes can produce clinically similar consequences. These complexities are bypassed in the simple mitochondrial disease model, *Dictyostelium discoideum* [20].

*D. discoideum* is one of 10 valuable non-mammalian models according to the USA National Institutes of Health [20,21]. Techniques such as genetic transformation [22], determination of gene copy numbers [23], gene knockdown by antisense RNA inhibition [24], GFP (green fluorescent protein) tagging of proteins, protein extraction, purification and immunodetection [25] and accessible measurement of diverse phenotypes [26] are well developed. This provides a readily exploited opportunity to manipulate HTRA2 expression levels at the molecular level and examine the consequences in vivo in *D. discoideum*.

Although the serine protease activity of HTRA2 has been reported to play a positive protective role in removing aberrant proteins in the mitochondria, it is unclear what level of the serine protease activity should be maintained in this process. Would elevated expression and activity of HTRA2 in the mitochondria cause any damage to cells? In a recently reported PD case, a P143A substitution in HTRA2 resulted in hyperphosphorylation (and presumably elevated activity) and was suggested to have contributed to PD pathology and mitochondrial dysfunction [27]. What would be the consequences if the serine protease activity of mitochondrial HTRA2 is removed? We confirm here that HTRA2 in *Dictyostelium* is localized in the mitochondria, from which location it plays a positive and/or protective role in regulating growth and development. Its knockdown causes phenotypic outcomes that are only partly reminiscent of other mitochondrial defects in this organism and are also distinct from those recently reported to result from loss of another PD-associated protein DJ-1 [28]. Despite its positive, cytoprotective roles, mitochondrial overexpression of wild type HTRA2 is lethal because of its proteolytic activity. Overexpression of a protease-dead mutant form is not lethal but faithfully phenocopies the effects of reduced expression. Mitochondrial respiration was not impaired either by HTRA2 knockdown or overexpression of protease-dead HTRA2.

## 2. Materials and Methods 

### 2.1. Plasmid Constructs

The HTRA2 antisense construct pPROF689 was created by subcloning a fragment of the *htrA* gene (763–1570 bp) into the vector pDNeo2 [29]. The HTRA2 overexpression construct pPROF691 was created by subcloning the full length *htrA* gene into the vector pPROF267 [28]. The construct pPROF692 was generated by insertion of the *htrA* gene with two nucleotide substitutions (T977G and T979A) into pPROF267 for expression of HTRA2^S300A^. The *htrA* gene without the stop codon was cloned in frame into pA15GFP to generate the construct pPROF694 for expression of HTRA2:GFP. For expression of HTRA2^S300A^:GFP, the construct pPROF695 was made by inserting in frame into pA15GFP the full length *htrA* without the stop codon and with the T977G and T979A substitutions.

### 2.2. *Dictyostelium discoideum* Strains and Culture Conditions

All experiments were conducted with *D. discoideum* parental strain AX2 and transformants derived from it as previously [30]. Strains HPF1191-HPF1205 carried multiple copies of the HTRA2 antisense inhibition construct pPROF689; strains HPF1220-HPF1231 had been transformed with the HTRA2 overexpression construct pPROF691; strains HPF1232-HPF1244 expressed multiple copies of the HTRA2^S300A^ construct pPROF692; for use as a positive control in GFP expression experiments HPF1245 and HPF1246 expressed a GFP-tagged DJ-1 fusion protein in the pA15GFP vector as described previously [28]; HPF1247-HPF1249 had been transformed with pPROF694, the HTRA2:GFP expression construct; HPF1250 and HPF1261 contained the construct pPROF695 which enabled expression of HTRA2^S300A^:GFP. 

The culture conditions of *D. discoideum* cells on solid and in liquid medium were described previously [28]. 

### 2.3. Molecular Biology Methods

Molecular manipulations were performed as described previously [28].

#### 2.3.1. Polymerase Chain Reaction Amplification and Cloning of *htrA*

A 2023 bp *htrA* sequence encoding the full length HTRA2 was amplified, cloned into pUC18 and subcloned into pPROF267 vector with primers HtrA2F (^5′^GCGAATTCTTCGAAATGATTCAATCTTCAATTAGAAAATG^3′^) and HtrA2R (^5′^GCGAATTCCTCGAGTTAAAAAATAGTTTTATTACTATTATC^3′^). The *htrA* gene without the stop codon and with two base substitutions was amplified and cloned into pUC18 vector and subcloned into pA15GFP for expression of HTRA2S300A:GFP with forward primer HtrA2(S300A)F (^5′^CCAGGTAATGCAGGTGGTCCAG^3′^) and reverse primer HtrA2(S300A)R (^5′^CTGGACCACCTGCATTACCTGG^3′^). To verify the existence of *htrA* in construct pPROF691, a fragment of 2325 bp including the A15-P promoter (302 bp) and full length *htrA* (2023 bp) was amplified using the primers A15PIF302 (^5′^GGATGGTGAAGATGTTCAAGC^3′^) and HtrA2R (^5′^GCGAATTCCTCGAGTTAAAAAATAGTTTTATTACTATTATC^3′^).

#### 2.3.2. Quantitative PCR and Quantitative Real Time-PCR

The construct copy numbers in *D. discoideum* transformants were quantitated by using iQ SYBR Green Supermix (Bio-Rad, Hercules, California, U.S.A.) and the messenger RNA (mRNA) was quantitated by iScript^TM^ One-Step RT-PCR Kit (Bio-Rad), which were described previously [28]. For both quatitative PCR (qPCR) and quantitative Real Time-PCR (qRT-PCR), results were normalized against the single copy gene or mRNA encoding the cytoskeletal protein, filamin. 

The primers were Fil443For (^5′^CCACAGAGATATTGGAGTTGCGTACC^3′^), Fil552Rev (^5′^CAACTCAACCAATGTGCCTGCCAA^3′^), Fil1588F (^5′^CCCTCAATGATGAAGCC^3′^), Fil1688R (^5′^CCATCTAAACCTGGACC^3′^), HtrA2qPCRF (^5′^ACGAGTTACATCCATTCTCTGCCG^3′^), HtrA2qPCRR (^5′^ACCATCATCAGCCACTGATACACC^3′^), HtrA2-mRNAF (^5′^CCACAAAGAGAAGTAACTGGTAGTGG^3′^), HtrA2-mRNAR (^5′^GGTTACCATCACCAATGTTTGATCCGC^3′^), GFP-qPCRF (^5′^CCATTACCTGTCCACACAATCT^3′^), GFP-qPCRR (^5′^TCCATGCCATGTGTAATCCC^3′^).

#### 2.3.3. Western Blotting

A crude protein lysate was prepared by lysing 5 × 106 growth phase amoebae on ice in 15 μL Laemmli buffer containing 1 μL 25× protease inhibitor cocktail (Roche, Basel, Switzerland). The sample was then boiled for 10 min before loading 300 μg (Bradford assay) of *D. discoideum* protein onto a 12% SDS-PAGE (Sodium Dodecyl Sulfate Polyacrylamide Gel Electrophoresis) gel for gel electrophoresis. The mini Trans-Blot Turbo^TM^ blotting apparatus (Bio-Rad) was used for western transfer to mini PVDF (polyvinylidene difluoride) membrane as recommended by the manufacturer’s manual. To detect the GFP, a rabbit anti-GFP polyclonal antibody (1:2000) conjugated with Alexa Fluor-647 (ThermoFisher Scientific, Waltham, Massachusetts, U.S.A.) was used. The result was scanned on a Storm 860^TM^ Phosphorimager (GE Healthcare, Chicago, Illinois, U.S.A.).

### 2.4. Transformation of *Dictyostelium discoideum* and Phenotypic Analysis

All transformants were obtained using the methods described previously [28] and then analysed phenotypically.

#### 2.4.1. Phenotypic Analysis of *Dictyostelium* Strains

The phenotypic studies including phototaxis, plaque expansion, phagocytosis, growth in liquid, pinocytosis and morphogenesis were conducted as described previously [28]:

- For phototaxis, sterile toothpicks were used to transfer small quantities of vegetative amoebae onto small patches on non-nutrient charcoal agar plates from the edges of growing plaques on *Enterobacter aerogenes* lawns [28].
- Plaque expansion rates were measured by inoculating amoebae at the centre of an *Escherichia coli* B2 lawn on normal agar plates and measuring plaque diameters twice daily for five days [28]. Expansion rates were determined using linear regression.
- Phagocytosis rates were measured as the rate of amoebal fluorescence increase in 20 mM pH 7 phosphate buffer containing *E. coli* cells expressing the red fluorescent protein DsRed. Fluorescence was measured over a 30 min period after harvest, washing and lysis of the amoebae [28].
- Growth in liquid was measured by inoculating 10^4^ exponentially growing cells/mL from HL-5 medium into fresh medium, incubating in shaken culture at 21 °C and counting cells twice daily for five days. Growth rates (h/generation) were determined by log-linear regression [28].
- Pinocytosis rates were determined as the rate of uptake over a 70 min period by 10^7^ vegetative amoebae/mL of 20 mg/mL fluorescein isothiocyanate (FITC)-dextran in HL-5. Aliquots of amoebae were harvested in Sorensen’s buffer (2 mM Na_2_HPO_4_.2H_2_0, 14.67 mM KH_2_PO_4_, pH 6.0), washed and lysed for fluorescence measurements [28].
- Morphogenesis was assayed by photography under dissecting microscopy of mature fruiting bodies formed in plaques on growth plates with *E. aerogenes* lawns [28].

#### 2.4.2. Seahorse Respirometry

The method was described previously [31]. Exponentially growing *Dictyostelium* amoebae were harvested, washed and resuspended in SIH assay medium (Formedium, Hunstanton, Norfolk, United Kingdom) supplemented with 20 mM sodium pyruvate and 5 mM sodium malate (pH 7.4). For each strain to be tested, 1 × 10^5^ cells were inoculated into each of eight Matrigel-coated wells in a 24-well assay plate for the Seahorse XFe^24^ Flux Analyser (Agilent Technologies, Santa Clara, California, U.S.A.) and allowed to attach for ca. 30 min. After the calibration and equilibration steps (ca. 20 min), measurements throughout the assay were conducted using cycles of 3 min mixing, 2 min wait and 3 min measurement time. The basal O_2_ Consumption Rate (OCR) was measured for three measurement cycles and this was followed by OCR measurements after sequential injections of 10 μM *N*,*N*’-dicyclohexylcarbodiimide (DCCD, adenosine triphosphate (ATP) synthase inhibitor (Sigma-Aldrich, St. Louis, Missouri, U.S.A.); six measurement cycles), 10 μM carbonyl cyanide 3-chlorophenol hydrazone (CCCP, protonophore, Sigma-Aldrich; three measurement cycles), 20 μM rotenone (Complex I inhibitor, Sigma-Aldrich; three measurement cycles), and either 10 μM antimycin A (Complex III inhibitor, Sigma-Aldrich; four wells, three measurement cycles) or 1.5 mM benzohydroxamic acid (Alternative Oxidase/AOX inhibitor, BHAM (benzohydroxamate), Sigma-Aldrich; four wells, three measurement cycles). The parental AX2 strain was included in every experiment in four wells (two for each of the final antimycin A and BHAM injections). From the measurements before and after each addition, the basal and maximum CCCP-uncoupled respiration rates were determined as well as the contributions to them of ATP synthesis, Complex I, Complex II and “nonmitochondrial” O_2_ consumption by other cellular oxidases and oxygenases.

## 3. Results

### 3.1. A Single Homologue of Human HTRA2 Is Encoded in the *Dictyostelium discoideum* Genome and Localized in the Mitochondria

**Figure 1:**
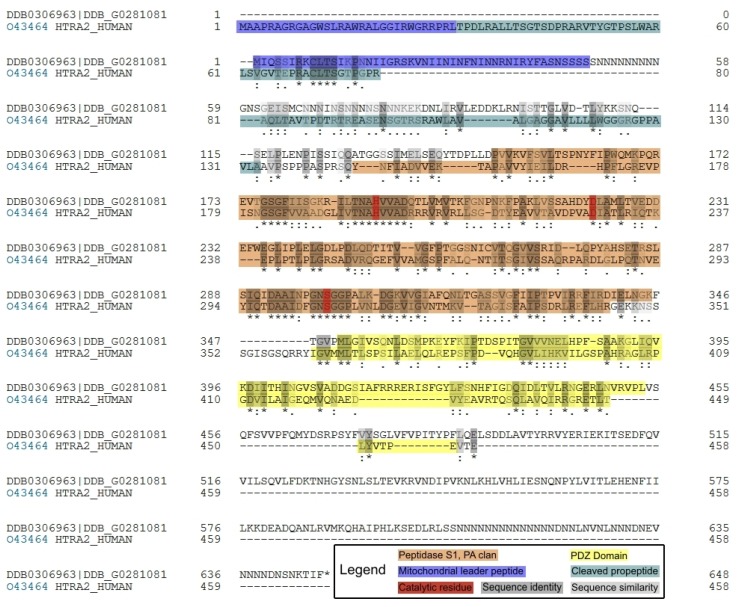
ClustalO sequence alignment of *Dictyostelium discoideum* and human HTRA2 sequences. The canonical HTRA2 protein in *Homo sapiens* contains 458 amino acids encoded by a gene 4152 bp long with eight exons and seven introns [33], whereas in *D. discoideum*, it consists of 647 amino acids encoded by a 2023 bp gene with one intron. The three essential catalytic residues (the catalytic triad) are highlighted in red, including the conserved catalytic serine, substituted with alanine in this and previous work [33]. The alignment was performed using ClustalO [34], the mitochondrial leader peptide was predicted using Mitoprot II [35] and the domains were identified using InterPro Scan [36]. The human protein includes a propeptide that is removed during processing to form the mature protein [37]. The *Dictyostelium* protein includes additional low complexity regions near the N- and C-termini. Such low complexity “additions” are common in *Dictyostelium* proteins.

A BLAST (basic local alignment search tool) search at dictyBase [32] using the protein sequence of human HTRA2 [33] as the query sequence identified a single homologous protein (which we designate HTRA2) encoded in the *D. discoideum* genome by a gene (accession number DDB_G0281081) we designate as *htrA,* full protein sequence alignment shown in (Figure 1). 

Human HTRA2 is reported to be localized in the mitochondria [37] and, to verify this predicted localization in *D. discoideum*, three mitochondrial prediction programs (MitoProt II [35], Predotar [38] and Helical Wheel [39]) were used to determine the likelihood of HTRA2’s mitochondrial localization. The results predict that, like its human counterpart, HTRA2 is localized to the mitochondria in *D. discoideum* (Figure 1, Figure S1, Table S1, Figure S2). As well as the predicted mitochondrial targeting signal at the N-terminus of the protein sequence, InterProScan analysis [36] detected a serine protease domain (residues 141–345) and PDZ protein-binding domain (residues 352–453) in the locations expected from the positions of the corresponding regions in the human protein (Figure 1).

**Figure 2:**
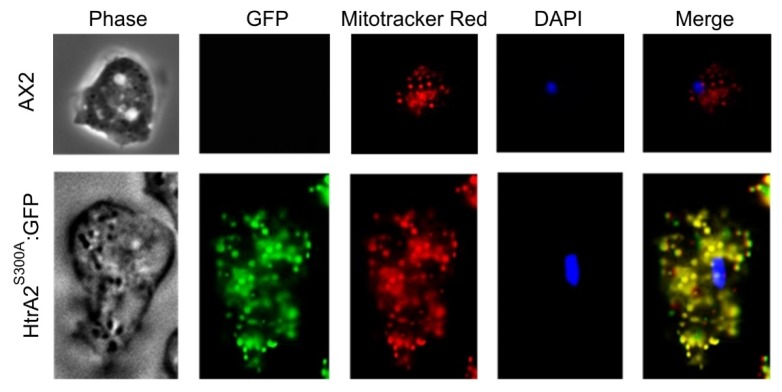
*Dictyostelium* HTRA2 is located in the mitochondria. The top panels show live AX2 cells and the bottom panels are live transformants expressing HTRA2^S300A^:GFP, a GFP-tagged protease-dead mutant form of HTRA2. PH: phase contrast of the *D. discoideum* cells, DAPI: 4′, 6-diamidino-2-phenylindole, used to stain nuclei, GFP: green fluorescence protein, used to detect HTRA2^S300A^:GFP, Mitotracker: Mitotracker Red, used to stain mitochondria, Merge: the overlap of all the images. The differences in size and numbers of stained mitochondria between the wild type and transformed cells are coincidental—both of these features vary significantly from cell to cell even within the same preparation (see examples in [28]).

Our attempts to confirm experimentally the mitochondrial localization of *Dictyostelium* HTRA2 by expressing wild type HTRA2 tagged at the C-terminus were unsuccessful (Section 3.5.1) and we tried but were unable to generate an anti-HTRA2 antibody that could be used in immunofluorescence microscopy. However, we did succeed in isolating transformants expressing a C-terminally GFP-tagged, protease-dead mutant (S_300_ substituted with alanine) form of the protein (Section 3.5.2). Epifluorescence microscopy revealed the fusion protein to be located in the mitochondria as expected (Figure 2). C-terminal GFP-tagging is a standard approach to tagging mitochondrial proteins because the mitochondrial targeting signal is normally (as in this case) a leader peptide at the N-terminus.

### 3.2. Creation of HTRA2 Knockdown Transformants

**Figure 3:**
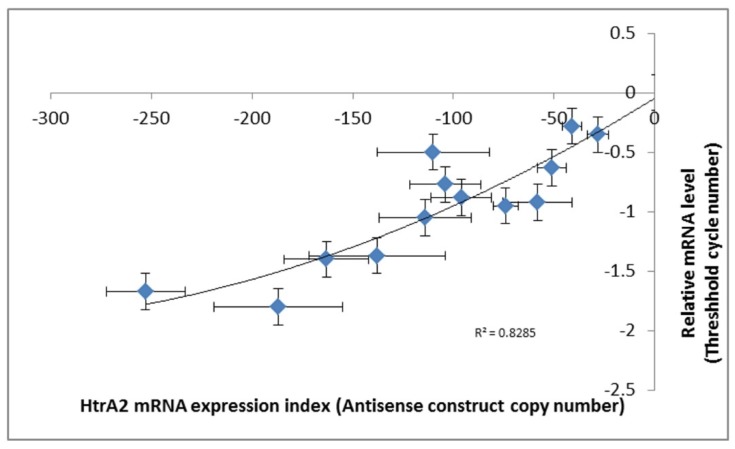
Expression of HTRA2 messenger RNA (mRNA) correlates with the copy numbers of the antisense-inhibition construct, pPROF689. The copy numbers of pPROF689 in HTRA2 antisense-inhibited transformants were measured by quantitative PCR (qPCR) and ranged from 28 to 252. The transcription of *htrA* was strongly correlated with the pPROF689 copy number (*p* = 6.2 × 10^−5^, quadratic regression, F test, *n* = 13). Error bars are standard errors from three independent experiments, each involving duplicate measurements. The previously established convention of using negative numbers for the antisense expression index was followed, so that *htrA* mRNA expression levels increase from left to right.

To study the phenotypic outcomes of loss of HTRA2 function in *Dictyostelium*, we created a HTRA2 antisense-inhibition construct (pPROF689) and used it to isolate multiple knockdown transformants of the parental *D. discoideum* strain AX2. The constructs insert randomly into the genome in a process accompanied by rolling circle replication which generates transformants, each with a unique number of copies of the construct so that each transformant has a different level of antisense inhibition [40]. The copy numbers of the inserted construct were determined by qPCR and the expression levels of the construct were measured by qRT-PCR. The relationship between *htrA* mRNA expression levels and the copy numbers of HTRA2 antisense construct are shown in (Figure 3). 

### 3.3. Mitochondrial HTRA2 Is Needed for Normal Morphogenesis and Growth, but Not for Phototaxis

**Figure 4:**
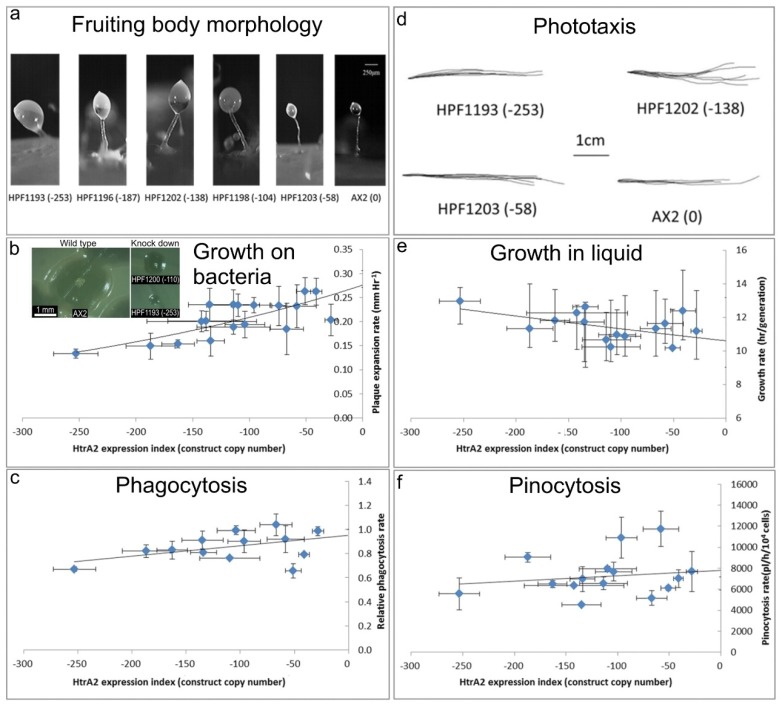
Phenotypic consequences of reduced HTRA2 expression.

In *D. discoideum*, mitochondrial respiratory dysfunction has been created by various genetic methods, and in each case the mitochondrially diseased strains presented consistent phenotypes, including impaired growth, phototaxis and morphology [30,41,42,43,44]. These included heteroplasmic knockout of nine different genes in the mitochondrial genome, knockout of a nuclear-encoded Complex I assembly factor (MidA) and knockdown of the nuclear-encoded mitochondrial chaperone (Chaperonin 60) required for folding of mitochondrial proteins [20]. If inhibition of HTRA2 causes mitochondrial respiratory dysfunction, it should generate the same phenotypes caused by *D. discoideum* mitochondrial respiratory disease. To verify this, the *D. discoideum* transformants containing HTRA2 antisense-inhibition construct were characterized phenotypically (Figure 4).

(a) Aberrant fruiting body morphology.

Wild type AX2 fruiting bodies have long and thin stalks, whereas the antisense transformants produced larger fruiting bodies with short, thick stalks. The severity of the defect correlates with decreasing HTRA2 expression level as indicated by the copy numbers of the HTRA2 antisense-inhibition construct (pPROF689—copy numbers in parentheses).

(b) Slower rate of plaque expansion on bacterial lawns.

Plaque sizes were measured over several days, the plaque expansion rates calculated by linear regression and then plotted against the expression index for HTRA2 (pPROF689 copy numbers). The plaque expansion rate of wild type AX2 was less than 0.3 mm hr^−1^. The plaque expansion rates decreased as the level of HTRA2 expression decreased. The quadratic regression was significant at *p* = 2.22 × 10^−4^ (F test, *n* = 19). Error bars are standard errors of the mean from four independent experiments. The inset shows as examples, single colonies of AX2 and two knockdown strains after five days incubation on streak dilution plates on lawns of *E. aerogenes* (construct copy numbers in parentheses).

(c) Normal rate of phagocytosis.

The phagocytosis rates of HTRA2 knockdown strains were measured as the rate of uptake of fluorescent *E. coli* DsRed cells and normalized against AX2. There was no significant correlation of phagocytosis rates with the HTRA2 expression level (*p* = 0.076, F test, *n* = 14). Error bars are standard errors of the mean from three independent experiments.

(d) Normal phototaxis by multicellular slugs.

The slug trails of parental strain AX2 and the HTRA2 antisense transformants containing different copy numbers of pPROF689 as indicated in brackets. The light source was located to the right of the figure.

(e) Slightly slower growth in liquid.

The knockdown strains grew at a slightly slower rate than AX2 (*p* = 0.0193, F test, *n* = 16). Error bars are standard errors of the mean from three independent experiments.

(f) Normal rate of pinocytosis.

The knockdown strains showed no significant reduction in pinocytosis rates (rate of uptake of FITC-containing medium) as the severity of the antisense inhibition increased (*p* = 0.5597, F test, *n* = 14). Error bars are standard errors of the mean from three independent experiments.

#### 3.3.1. Fruiting Body Morphology Is Regulated by HTRA2 

Mitochondrial dysfunction in *D. discoideum* results in defective fruiting body morphology– culminants have shorter and thicker stalks and this phenotype is mediated by chronic activation of AMPK [20,41,42]. This phenotype was examined in the HTRA2 antisense-inhibited transformants to determine if it would phenocopy mitochondrial dysfunction. The morphology of AX2 and HTRA2 antisense-inhibited transformants was examined after growth and development on bacterial lawns on nutrient agar (SM) plates. (Figure 4a) shows that the reduction in HTRA2 expression resulted in aberrant fruiting bodies with shorter and thicker stalks compared to AX2. The severity of this abnormal morphology was correlated with the copy numbers of the HTRA2 antisense-inhibition construct (pPROF689). It resembles the morphology of mitochondrially diseased *D. discoideum* strains, a result consistent with the possibility that HTRA2 loss impairs mitochondrial respiratory function.

#### 3.3.2. Knocking Down HTRA2 Expression Inhibits Plaque Expansion in *Dictyostelium discoideum* but Does Not Affect Phagocytosis Rates

Another defective phenotype that characterizes mitochondrial disease in *Dictyostelium* is impaired plaque expansion or growth on *E. coli* B2 lawns. HTRA2 antisense-inhibited strains displayed decreased plaque expansion rates that correlated with the construct copy number (Figure 4b). At the highest copy numbers of the antisense construct, the plaque expansion rate was approximately halved. This reduced rate of growth on bacterial lawns could not be explained by a defect in phagocytosis (Figure 4c). 

#### 3.3.3. Axenic Growth Is Affected Only Slightly and Pinocytosis Is Unaffected by HTRA2 Expression Levels

Laboratory strains of *D. discoideum* are able to grow on solid media and also axenically in liquid media. To determine if HTRA2 plays a role in growth in liquid media (axenic growth), AX2 and the knockdown strains were inoculated into sterilized HL-5 and the generation time was measured. The results showed that the generation times of HTRA2 antisense transformants are affected only slightly by reduced expression of HTRA2, with generation times being only about 20% longer even at the highest copy numbers of the antisense construct (Figure 4e). Growth in liquid medium by *Dictyostelium* cells is dependent on nutrient uptake by pinocytosis and when this was measured we found no significant effect of HTRA2 expression on the rates of pinocytosis (Figure 4f). This differs from the phenotype displayed by mitochondrially diseased *D. discoideum* strains whose growth typically is dramatically slower in liquid medium, although unaccompanied by corresponding defects in pinocytosis [20]. 

#### 3.3.4. HTRA2 Expression Levels Have No Effect on Phototaxis

The photosensory signalling pathway in *D. discoideum* is sensitive to mitochondrial respiratory dysfunction so that, with one exception, all tested genetic causes of mitochondrial dysfunction result in impaired orientation during slug phototaxis towards a lateral light source [20]. HTRA2 is a nuclear-encoded mitochondrial protein, and its inhibition may affect mitochondrial function. Therefore, we tested the phototactic ability of the HTRA2 antisense-inhibited transformants and found that the antisense transformants showed accuracies of phototaxis resembling the wild type AX2 (Figure 4d). This result is not consistent with HTRA2 knockdown causing impairment of mitochondrial respiratory function.

### 3.4. Mitochondrial Respiration Is Not Significantly Affected by Knockdown of HTRA2

**Figure 5:**
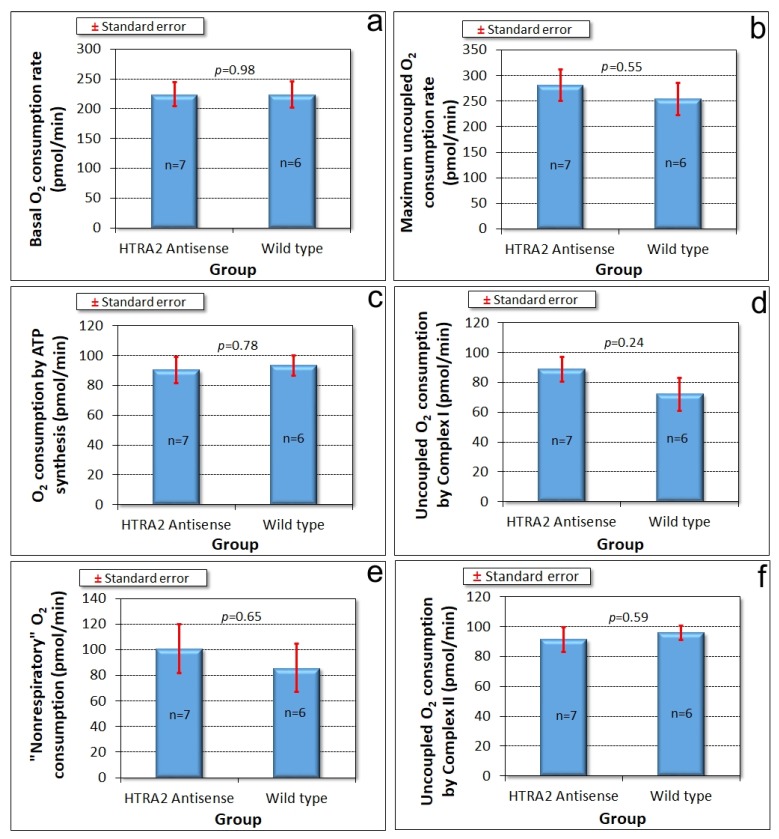
Mitochondrial respiration is unaffected by HTRA2 knockdown. Seahorse respirometry was conducted in the indicated number (n) of independent experiments on wild type (AX2) cells and cells of four different HTRA2 knockdown strains (antisense construct copy numbers ranging from 96 to 163). Basal respiration (**a**) and its components attributable to adenosine triphosphate (ATP) synthesis (**c**) and “nonmitochondrial” respiration (**e**) were unaffected. The maximum respiration rate by carbonyl cyanide 3-chlorophenol hydrazone (CCCP)-uncoupled mitochondria (**b**) and its components attributable to Complex I (**d**) and Complex II activity (**f**) were not significantly affected. The copy number of the antisense construct had no significant effect on any of the parameters of respiration (Pearson correlation coefficient, n = 13, *p* > 0.05).

The foregoing results showed that reduced levels of HTRA2 expression cause a pattern of phenotypes that exhibits only some of the defects typically observed in mitochondrially diseased *Dictyostelium* strains, namely aberrant morphogenesis and reduced growth rates, unaccompanied by defects in endocytosis. However, there is no defect in phototaxis and the defect in growth in liquid medium is only mild. These differences suggest that although HTRA2’s role in the mitochondria is positive and protective, its loss evokes cytopathological mechanisms that differ from the respiratory defects typical of mitochondrial disease. To verify that HTRA2 loss does not cause mitochondrial respiratory defects, we used Seahorse respirometry as previously described [31] to measure mitochondrial respiratory activity directly. The results showed that HTRA2 knockdown does not cause any significant loss of mitochondrial respiratory function (Figure 5).

### 3.5. Overexpression of HTRA2 Is Toxic to *Dictyostelium discoideum* Cells and This Toxicity Depends on Its Serine Protease Activity

#### 3.5.1. *Dictyostelium* Transformants Overexpressing Wild Type HTRA2 Cannot Be Isolated

To investigate the positive, protective role of HTRA2 further, we attempted to ectopically overexpress functional wild type HTRA2 in *Dictyostelium* cells. We created an HTRA2 overexpression construct (pPROF691) for expression of the full length wild type *htrA* gene under the control of the actin-15 promoter (A-15P). This was used to transform the parental strain AX2. These transformants were very difficult to isolate, averaging less than 10 per experiment, compared to hundreds in a single more typical transformation. After multiple experiments, 50 transformants were isolated and purified. PCR assays showed that only about half (26) of these contained the full length recombinant *htrA* gene. In the remainder, the recombinant gene was either undetectable by PCR or contained large deletions (Text S1, Figure S3). Of those transformants that contained the full length recombinant *htrA*, half had unusually low numbers of copies of the *htrA* amplicon, not very different from the single endogeneous copy of the gene (Text S1–3, Table S2). Three of the ca. 25% of transformants with moderate copy numbers of the the full length recombinant gene were studied further and shown to contain mutations in *htrA* (single base substitutions or insertions causing reading frame shifts and truncation of the protein) (Text S3, Figure S4). In combination with the large deletions demonstrated in most transformants, and the low copy numbers in most of the remainder, the mutations in these sequenced cases suggest that the serine protease activity of HTRA2 may be lethal in overexpression transformants because of uncontrolled proteolysis. The only survivors were the small fraction of transformed cells in which the *htrA* gene was poorly expressed or had been mutated or deleted.

#### 3.5.2. *Dictyostelium discoideum* HTRA2: GFP Fusion Transformants Do Not Express Functional GFP Fusion Protein

Not only were we unable to obtain transformants ectopically overexpressing the wild type HTRA2, we also were unable to isolate overexpressers of the wild type protein tagged at the C-terminus with GFP. Our intent had been to verify the subcellular localization of HTRA2. For this purpose, a construct expressing GFP-tagged HTRA2 (pPROF694) was transformed into wild type *D. discoideum* AX2 and analysed with fluorescent microscopy. We did obtain transformants, but there was no GFP fluorescence in any of them (e.g., Figure S5). 

**Figure 6:**
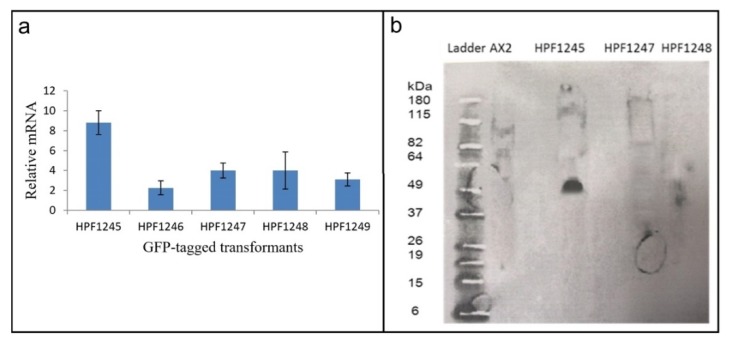
GFP mRNA is expressed in HTRA2: GFP fusion transformants, but the protein is not. HTRA2: GFP fusion transformants were isolated and shown to contain the full length *htrA* and GFP sequences (Figure S7). The GFP mRNA was expressed in these transformants but the protein was not. (**a**) Transcription of GFP in DJ-1: GFP and HTRA2: GFP transformants. HPF1245 and HPF1246 are the DJ-1: GFP expression transformants and HPF1247, HPF1248 and HPF1249 are the HTRA2: GFP transformants. The GFP mRNA was measured and normalized against filamin. GFP coding sequence was transcribed in all GFP-tagged *D. discoideum* transformants. Error bars are standard errors of the mean from duplicate measurements. (**b**) Expression of GFP fusion protein in DJ-1:GFP and HTRA2:GFP transformants. The Western blot includes protein from AX2 (negative control), HPF1245 expressing GFP-tagged DJ-1 (52 kDa) (positive control), and HPF1247 and HPF1248 containing constructs for expressing GFP-tagged HTRA2. No GFP could be detected in the HTRA2:GFP transformants. The amount of protein loaded in each well was 300 μg (Bradford assay).

To determine why the transformants displayed no fluorescence, the presence of the GFP and *htrA* sequences were verified by PCR. The results showed that both the *htrA* and the GFP portions of the HTRA2:GFP fusion gene in the construct (pPROF694) were present in their entirety in the transformants (Figure S6). We therefore determined if the GFP sequence in these transformants was expressed at both the RNA (qRT-PCR) (Figure 6a) and protein (Western blot) (Figure 6b) level. 

RNA was extracted from three HTRA2:GFP fusion transformants and from two DJ-1: GFP transformants [28] which were to be used as positive controls (as these do display GFP fluorescence). qRT-PCR was carried out to check expression of the GFP portion of the fusion mRNA using filamin as a reference gene [45]. The result showed that the recombinant GFP fusion gene was transcribed into mRNA (Figure 6a). To determine if the mRNA was translated, a Western blot was performed using an anti-GFP antibody. The blot showed that the GFP protein was not expressed in the HTRA2:GFP transformants, but was expressed in the DJ-1:GFP transformant [28] used as a positive control (Figure 6b). In view of the apparent toxicity of the wild type HTRA2, these results suggest that the GFP portion of the fusion protein was not expressed because of mutations and premature translational stops in the upstream HTRA2 portion of the protein.

### 3.6. A Protease-Dead Mutant (S300A) of Dictyostelium HTRA2 Can Be Expressed in *Dictyostelium discoideum*

The foregoing results suggested strongly that overexpression of active HTRA2 is toxic to *D. discoideum* cells, perhaps due to the uncontrolled serine protease activity. To confirm if this is true, an essential serine residue in the catalytic site of *D. discoideum* HTRA2 was selected and mutated to ablate its serine protease activity. Suzuki et al. [35] showed that human HTRA2 contains two main domains: the N-terminal serine protease domain which prevents the accumulation of misfolded proteins and the C-terminal PDZ domain which modulates the protease activity. According to Polgár [46], the trypsin-like proteases contain a catalytic triad of serine, histidine and aspartic acid residues that exhibit similar spatial arrangements in the active site. The serine protease domain of HTRA2 adopts the same fold as trypsin including the essential, highly conserved, catalytic serine (S306 in humans, S300 in *D. discoideum*) (Figure 1) [47,48]. Others have shown that S306 in the mammalian protein is essential for the proteolytic and protective activities of HTRA2. Therefore, we created a protease-dead mutant of the *Dictyostelium* HTRA2. 

To remove the serine protease activity of *D. discoideum* HTRA2, serine 300 (Figure 1) was replaced by alanine and the construct pPROF692 expressing the mutant form of the protein, HTRA2^S300A^, was transformed into the wild type *D. discoideum* AX2. A total of 280 transformants were obtained from a single (typical) transformation. To determine if the full HTRA2^S300A^ sequence was present in these transformants, the genomic DNA (gDNA) of 50 randomly chosen transformants was extracted and used as the template to amplify the full length recombinant gene. The results showed that all 50 tested transformants contained full length gene encoding HTRA2^S300A^, in stark contrast to the earlier results with the wild type HTRA2. Quantitative PCR showed that the copy numbers ranged from 42 to 312, a much more typical range for this kind of experiment. The expression of HTRA2^S300A^ mRNA was also measured using qRT-PCR and was strongly correlated with the construct copy number as expected (Figure S7). Taking all of the results for overexpression of the wild type and mutant forms of HTRA2 together, we conclude that wild type HTRA2 cannot be overexpressed in *Dictyostelium* due to the toxicity caused by high levels of protease activity. 

### 3.7. Phenotypic Analysis of HTRA2^S300A^ Transformants

The inability to overexpress HTRA2 in *D. discoideum* was due to the activity of the serine protease domain. By mutating the essential catalytic serine S_300_ in this domain, we were able to obtain overexpression transformants. When we fused this mutant form of the protein to GFP, we were also able to obtain recombinant GFP-expressing forms that confirmed the mitochondrial localization of the protein (Figure 2). Since HTRA2 is reported to exert a protective effect in the mitochondria, it was possible that overexpression of HTRA2^S300A^ would enhance this protective role. This would indicate that the protective functions of the protein in the mitochondria are not dependent on the protease activity. However, others have reported that HTRA2’s protective functions are lost when the protease activity is ablated. 

**Figure 7:**
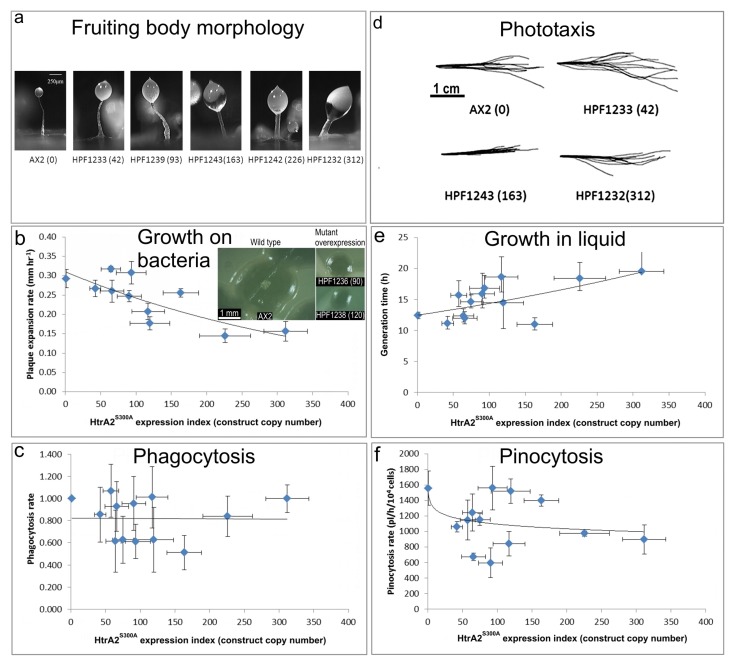
Phenotypic consequences of protease-dead HTRA2^S300A^ overexpression.

HTRA2 is a homotrimeric protease whose three PDZ protein-binding domains recruit a limited range of target substates and, upon binding them, activate the catalytic protease domain. Ectopic overexpression of a protease-dead form of the protein could result in competitive inhibition of the native wild type protein that is still expressed from the endogeneous *htrA* gene. This could be a result of competition for substrates and/or formation of inactive mutant/wild type heterotrimers. In either case, the overexpression of the protease-dead protein should phenocopy knockdown of expression of the endogeneous gene. To investigate whether this is so, we determined the phenotypes of transformants overexpressing HTRA2^S300A^ (Figure 7). 

(a) Aberrant fruiting body morphology.

The stalks of HTRA2^S300A^ transformant fruiting bodies were thick and short compared to the thin and long stalk of the AX2 fruiting body. This phenotype became more severe with an increase of HTRA2^S300A^ expression levels indicated by increasing copy numbers of pPROF692 in parentheses after the strain names of the individual transformants.

(b) Slower rate of plaque expansion on bacterial lawns. 

The plaque expansion rate of AX2 was 0.29 mm hr^−1^. Compared to AX2, 80% of transformants containing mutated HTRA2 displayed slower plaque expansion rates and the plaque expansion rates reduced with increasing HTRA2^S300A^ expression levels (regression of plaque expansion rate against pPROF692 (HTRA^2S300A^) copy numbers highly significant: *p* = 0.00396, F test, *n* = 11). Error bars are standard errors of the mean from four replicate measurements. The inset shows as examples, single colonies of AX2 and two HTRA2^S300A^ overexpression strains after five days incubation on streak dilution plates on lawns of *E. aerogenes* (construct copy numbers in parentheses).

(c) Normal rate of phagocytosis.

The phagocytosis rates of the transformants were not significantly affected by the HTRA2^S300A^ expression levels (regression not significant: *p* = 0.982, F test, *n* = 13). Error bars are standard errors of the mean from three independent experiments.

(d) Normal phototaxis by multicellular slugs.

Slugs of AX2 and transformants containing different numbers of copies of the HTRA2^S300A^ expression construct (shown in parentheses) migrated to the light source which was to the right of the figures. Like the parental AX2 slugs, the slugs of the transformants displayed normal phototaxis.

(e) Slightly slower growth in liquid.

The average generation time of HTRA2^S300A^ transformants was 15.6 + 0.9 h, significantly longer than the generation time of AX2 (10.4 + 2.6 h). The generation times of HTRA2^S300A^ transformants were weakly correlated with mHTRA2 expression levels as indicated by the copy numbers of pPROF692 expressing HTRA2^S300A^ and the correlation was statistically significant (Pearson correlation coefficient *ρ* = 0.58, *p* = 0.03, *n* = 11). Error bars are standard errors of the mean from three independent experiments.

(f) Normal rate of pinocytosis.

The pinocytosis rates of HTRA^2S300A^ overexpression transformants decreased slightly with increased mutant HTRA2 expression levels as indicated by increasing pPROF692 copy numbers, but the regression was not significant compared to wild type AX2 (Pearson correlation coefficient *ρ* = 0.23, *p* = 0.22, *n* = 13; log-linear regression not significant at *p* = 0.209, F test, *n* = 13). Error bars are standard errors of the mean from three independent experiments.

#### 3.7.1. Overexpression of Protease-Dead HTRA2 Resulted in Defective Fruiting Body Morphology 

Knockdown of endogeneous HTRA2 expression caused the formation of abnormal fruiting bodies. To determine if morphogenesis was affected similarly by overexpression of HTRA2^S300A^, the fruiting body morphology of the HTRA2 transformants was examined. The mutant overexpression strains had altered morphologies with larger sori and shorter, thicker stalks. The severity of this defect correlated with the HTRA2^S300A^ expression index (Figure 7a). This is similar to the defects resulting from HTRA2 knockdown and reminiscent of what occurs in mitochondrially diseased *Dictyostelium* strains, supporting the possibility that overexpression of mutant HTRA2 may result in altered mitochondrial function. 

#### 3.7.2. Plaque Expansion Was Inhibited but Phagocytosis Was Unaffected by Overexpression of HTRA2^S300A^

The growth of HTRA2^S300A^ expression strains on *E. coli* B2 lawn was measured and the results show that the plaque expansion rates decreased with increased expression levels of protease-dead HTRA2 (Figure 7b). To determine if this was due to a decreased ability to take up nutrients, phagocytosis rates were measured and plotted against the expression index (copy numbers) of HTRA2^S300A^. The results show that phagocytosis rates were unaffected in the HTRA2^S300A^ transformants (Figure 7c). This implies that the growth defect is not due to impaired phagocytosis, as was also the case for the HTRA2 knockdown strains.

#### 3.7.3. Growth in Liquid Is Slower While Pinocytosis Is Not Significantly Inhibited by Overexpression of HTRA2^S300A^

To determine if overexpression of protease-dead HTRA2 also affected axenic growth, the growth rates of the HTRA2^S300A^ transformants were measured in liquid media. Their generation times were calculated and plotted against the HTRA2^S300A^ expression index. The generation time of HTRA2^S300A^ transformants did increase and the severity of this defect was correlated with the HTRA2 expression index (Figure 7e).

To determine if there was a defect in the transformants’ ability to take up liquid nutrients, the pinocytosis rates were measured. The (Figure 7f) shows that although the HTRA2^S300A^ transformants appeared to exhibit slightly slower pinocytosis rates, the effect was not statistically significant. 

#### 3.7.4. Phototaxis and Mitochondrial Respiration Are Unaffected by Overexpression of Protease-Dead HTRA2^S300A^

**Figure 8:**
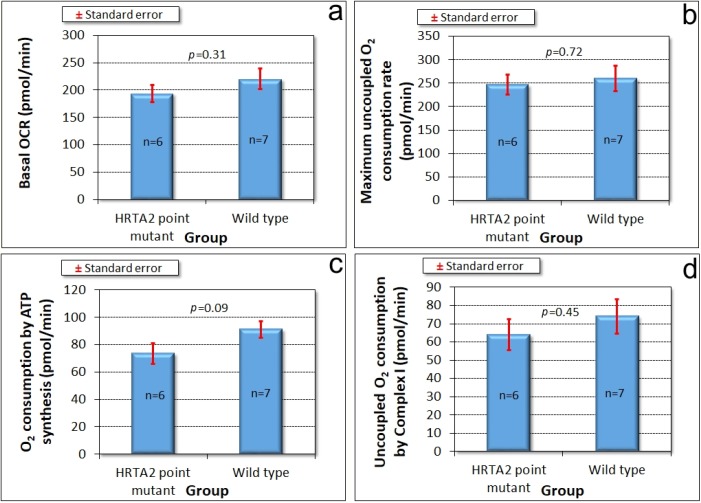

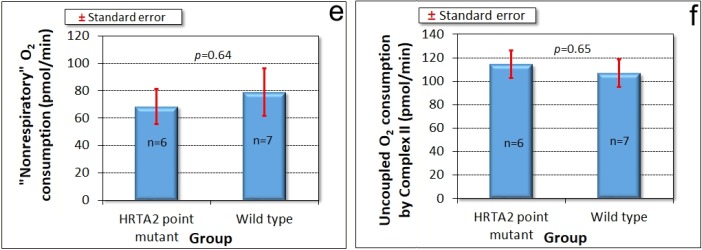
Mitochondrial respiration is unaffected by overexpression of protease-dead HTRA2^S300A^. Seahorse respirometry was conducted in the indicated number (n) of independent experiments on wild type (AX2) cells and cells of four different HTRA2^S300A^ overexpression strains (antisense construct copy numbers ranging from 90 to 312). Basal respiration **(a)** and its components attributable to ATP synthesis **(c)** and “non-mitochondrial” respiration **(e)** were unaffected. The maximum respiration rate by CCCP-uncoupled mitochondria **(b)** and its components attributable to Complex I **(d)** and Complex II **(f)** were also not significantly altered. The copy number of the HTRA2^S300A^ overexpression construct had no significant effect on any of the parameters of respiration (Pearson correlation coefficient, n = 13, *p* > 0.05).

Amoebae of AX2 and HTRA2^S300A^ overexpression transformants were inoculated onto charcoal agar plates without any food supply, allowed to form slugs and migrate towards a lateral light source. The slug trails were then blotted onto PVC (Polyvinyl chloride) discs, stained with Coomassie Brilliant Blue R and their trails were digitized. (Figure 7d) shows that phototaxis was not affected by overexpression of the mutant protease. As with the knockdown strains, the absence of a phototaxis defect is not consistent with an impairment of mitochondrial respiration and the absence of a mitochondrial respiratory defect was directly confirmed using Seahorse respirometry (Figure 8).

## 4. Discussion

The results reported here suggest that *Dictyostelium* HTRA2 is located in the mitochondria, from which location it plays a positive, cytoprotective role. We were able to demonstrate experimentally the subcellular location only of the GFP-tagged, overexpressed HTRA2^S300A^. However, there is no reason to expect that the wild type protein would localize differently, especially since it contains a recognizable N-terminal mitochondrial leader peptide and its human homologue has been reported to be mitochondrial [37]. This being so, cytopathology caused by genetic loss or reduction of HTRA2 activity constitutes a mitochondrial disease and as such has provided some of the evidence that Parkinson’s disease involves mitochondrial defects. However, the effects of HTRA2 loss on mitochondrial function remain unclear. For example, Yun et al. [19] suggested that HTRA2 did not act downstream of PINK1 in *Drosophila* as HTRA2 knockout mutants did not display the defective mitochondrial integrity and dynamics observed in PINK1 null mutants. Uncertainties regarding the cytopathological roles of HTRA2 mutations may result from the complexities of mitochondrial disease. Alternatively, they may be due to different experimental conditions (such as whether or not oxidative stress was applied) or different phenotypes being analyzed (such as mitochondrial integrity and dynamics, degeneration in direct flight muscles, mitochondrial morphology, male sterility and so on). To investigate the cytopathological roles of mitochondrial HTRA2, a model providing consistent phenotype-genotype correlations in mitochondrial dysfunction would be an advantage.

The *Dictyostelium* model provides such an advantage, so in this work we asked whether the cytopathological outcomes of HTRA2 loss in *Dictyostelium* are consistent with and caused by impaired mitochondrial respiration. We found that genetically impaired HTRA2 function (by antisense inhibition or presumed competitive inhibition by an ectopically overexpressed mutant form) had no effect on phototaxis, but caused defects in morphogenesis and growth (particularly on bacterial lawns), unaccompanied by corresponding defects in endocytosis. The slow plaque expansion rates on bacterial lawns could be due to slow growth or impaired amoeboid motility or both. This pattern of phenotypes is only partly reminiscent of the typical outcomes of mitochondrial respiratory disease in *Dictyostelium*, which are caused by chronic hyperactivity of the energy stress-sensing protein kinase AMPK [20,41,42,43,44]. This produces a variety of AMPK-mediated phenotypes, including impaired phototaxis, morphogenesis and equally severe defects in growth both on bacteria and in liquid medium [20]. The growth defects are not caused or accompanied by corresponding defects in endocytosis [20]. 

Since HTRA2 knockdown does not faithfully phenocopy mitochondrial respiratory disease, the defects it causes seemed unlikely to result from impaired mitochondrial respiration. Direct measurement of mitochondrial respiratory activity confirmed that mitochondrial respiration was not inhibited either by HTRA2 knockdown, or by overexpression of the inhibitory, protease-dead HTRA2^S300A^. Despite its mitochondrial location, HTRA2 is thus not essential for normal mitochondrial respiratory activity, suggesting that its protective role may instead be directed primarily towards other mitochondrial functions. The possibilities could include lipid, amino acid or iron metabolism as well as signalling by small molecules such as Ca^2+^, succinate or reactive oxygen species (ROS). In all of these cases, the resulting dysregulation could in turn account (indirectly) for the impaired growth that was observed in the antisense transformants.

HTRA2 loss also partly phenocopies the loss of another PD-associated protein, DJ-1, which also has no effect on phototaxis, but severely impairs growth on bacteria and only slightly impairs growth in liquid [28]. In the case of DJ-1 loss, however, the growth defects are accompanied and presumably caused by a corresponding deficiency in the requisite endocytic pathway. These results suggest distinct, but intersecting pathways underlying the cytopathology of different forms of mitochondrial disease and of PD. It will be of interest in the future to determine if chronic AMPK hyperactivity contributes to the aberrant phenotypes in cells with reduced HTRA2 activities.

Despite its positive protective role in the mitochondria, we found that wild type HTRA2 is cytotoxic when ectopically overexpressed. This was revealed by the difficulty of obtaining HTRA2 overexpression transformants, by the unusually low copy numbers of most transformants that we were able to obtain, by the mutations in the HTRA2 coding sequence in those few transformants with higher copy numbers and by the absence of expression and fluorescence of GFP in those HTRA2:GFP transformants that could be isolated. Mutation of the serine protease domain overcame this, so that many transformants expressing HTRA2^S300A^ were obtained and several were further studied. 

It has been suggested that, as a serine protease, HTRA2 plays its crucial protective role by contributing to protein quality control in the mitochondria [12,49,50]. This protective proteolytic function might also apply to *D. discoideum* and, as has been suggested for other organisms, could explain why HTRA2 loss causes phenotypic defects. On the other hand, when HTRA2 was overexpressed, the excess HTRA2 may have degraded essential proteins in the mitochondria thereby initiating cell death. Alternatively, HTRA2 overexpression might have caused an imbalance in HTRA2 levels inside and outside the mitochondria. Although HTRA2 usually localizes to the mitochondria, its overexpression could have resulted in incomplete import of the protein into the mitochondria or its partial release from the mitochondria to the cytosol where it may have initiated cell death. However, this possibility is not supported by the fact that overexpressing the GFP-tagged, protease-dead HTRA2^S300A^ did not reveal any significant failure of targeting of the overexpressed protein to the mitochondria. 

The mitochondrial localization of HTRA2^S300A^, even when overexpressed, is consistent with findings in other organisms and confirms the in silico predictions of a mitochondrial location for this protein. Thus, the serine protease activity of HTRA2 exerts a double-edged function in the mitochondria—it usually protects the cells, presumably by removing denatured mitochondrial proteins, but its hyperactivity in the mitochondria is also cytotoxic. One implication is that, while loss of function mutations in HTRA2 can cause cellular pathology, mutations that upregulate its protease activity could also do so, albeit by a different mechanism. For example, a P143A substitution results in hyperphosphorylation (and presumably elevated activity) as well as increased neurotoxicity of human HTRA2 [27]. This has been suggested to have contributed to PD pathology and mitochondrial dysfunction in a Taiwanese patient carrying this allele [27]. The PDZ domain of HTRA2 is required for keeping the protease activity of the protein in check and for recruitment of the correct substrates [48]. Its loss could produce less discriminating, elevated proteolytic activity that would also cause mitochondrial dysfunction. Both of the phosphorylatable serines in HTRA2 that have been implicated in PD (S142 and S400) are conserved in *Dicyostelium* (Figure 1). It would be valuable in future work to study their roles in this model.

In mammalian cells, HTRA2 has been reported to translocate from the mitochondria to the cytosol under stress conditions, where it initiates both caspase-dependent and -independent cell death pathways [2]. However, our results suggest that overactive HTRA2 is lethal to cells even in its original mitochondrial location. This is consistent with the recent report that overexpressed, wild type HTRA2 is targeted to the mitochondria, causing a motor defect and cell death in the brains of transgenic mice [51]. Like us, these authors also found that a PD-associated point mutant form of HTRA2 (HTRA2^G399S^) was targeted to the mitochondria and caused a dominant loss of function when overexpressed. It will be of interest in future work to determine if *Dictyostelium* HTRA2 also relocates to the cytosol under stress conditions and whether that has different cytopathological consequences. 

## Supplementary Materials

The following are available online at http://www.mdpi.com/2073-4425/9/7/355/s1, Text S1: Only half of the *D. discoideum* HTRA2 overexpression transformants contained the full length *htrA* gene in the overexpression construct (pPROF691), Text S2: Copy numbers are low in half of the transformants containing the full length *htrA* gene in the overexpression construct (pPROF691), Text S3: The recombinant *htrA* gene from higher copy number HTRA2 overexpression transformants is mutated, Figure S1: Prediction of HTRA2’s subcellular localization using MitoProt II., Figure S2: Prediction of HTRA2’s subcellular localization using a Helical Wheel plot, Figure S3: Amplification of *htrA* from HTRA2 overexpression transformants, Figure S4: Partial sequence of HTRA2 from HTRA2 overexpression transformants, Figure S5: GFP fluorescence of HTRA2:GFP fusion (HGFP) transformants, Figure S6: HTRA2:GFP fusion transformants with full length *htrA* and GFP sequences can be isolated, Figure S7: Expression of HTRA2^S300A^ mRNA correlates with the number of copies of mutated *htrA*, Table S1: Prediction of HTRA2’s subcellular localization using Predotar, Table S2: Copy numbers of htrA in HTRA2 overexpression transformants.

## References

[1] Hegde R., Srinivasula S.M., Zhang Z., Wassell R., Mukattash R., Cilenti L., DuBois G., Lazebnik Y., Zervos A.S., Fernandes-Alnemri T. (2002). Identification of Omi/HTRA2 as a mitochondrial apoptotic serine protease that disrupts inhibitor of apoptosis protein-caspase interaction. J. Biol. Chem..

[2] Vande Walle L., Lamkanfi M., Vandenabeele P. (2008). The mitochondrial serine protease HTRA2/Omi: An overview. Cell Death Differ..

[3] Martins L.M., Iaccarino I., Tenev T., Gschmeissner S., Totty N.F., Lemoine N.R., Savopoulos J., Gray C.W., Creasy C.L., Dingwall C., Downward J. (2002). The serine protease Omi/HTRA2 regulates apoptosis by binding XIAP through a reaper-like motif. J. Biol. Chem..

[4] Cilenti L., Kyriazis G.A., Soundarapandian M.M., Stratico V., Yerkes A., Park K.M., Sheridan A.M., Alnemri E.S., Bonventre J.V., Zervos A.S. (2005). Omi/HTRA2 protease mediates cisplatin-induced cell death in renal cells. Am. J. Physiol. Ren. Physiol..

[5] Liu Z., Li H., Derouet M., Berezkin A., Sasazuki T., Shirasawa S., Rosen K. (2006). Oncogenic ras inhibits anoikis of intestinal epithelial cells by preventing the release of a mitochondrial proapoptotic protein Omi/HtrA2 into the cytoplasm. J. Biol. Chem..

[6] Strauss K.M., Martins L.M., Plun-Favreau H., Marx F.P., Kautzmann S., Berg D., Gasser T., Wszolek Z., Müller T., Bornemann A. (2005). Loss of function mutations in the gene encoding Omi/HtrA2 in Parkinson’s disease. Hum. Mol. Genet..

[7] Bogaerts V., Nuytemans K., Reumers J., Pals P., Engelborghs S., Pickut B., Corsmit E., Peeters K., Schymkowitz J., De Deyn P.P. (2008). Genetic variability in the mitochondrial serine protease HTRA2 contributes to risk for Parkinson disease. Hum. Mutat..

[8] Gulsuner H.U., Gulsuner S., Mercan F.N., Onat O.E., Walsh T., Shahin H., Lee M.K., Dogu O., Kansu T., Topaloglu H. (2014). Mitochondrial serine protease HTRA2 p.G399S in a kindred with essential tremor and Parkinson disease. Proc. Natl. Acad. Sci. USA.

[9] Kawamoto Y., Kobayashi Y., Suzuki Y., Inoue H., Tomimoto H., Akiguchi I., Budka H., Martins L.M., Downward J., Takahashi R. (2008). Accumulation of HtrA2/Omi in neuronal and glial inclusions in brains with α-synucleinopathies. J. Neuropathol. Exp. Neurol..

[10] Zeth K. (2004). Structural analysis of DegS, a stress sensor of the bacterial periplasm. FEBS Lett..

[11] Wilken C., Kitzing K., Kurzbauer R., Ehrmann M., Clausen T. (2004). Crystal structure of the DegS stress sensor: How a PDZ domain recognizes misfolded protein and activates a protease. Cell.

[12] Moisoi N., Klupsch K., Fedele V., East P., Sharma S., Renton A., Plun-Favreau H., Edwards R.E., Teismann P., Esposti M.D. (2009). Mitochondrial dysfunction triggered by loss of HtrA2 results in the activation of a brain-specific transcriptional stress response. Cell Death Differ..

[13] Plun-Favreau H., Klupsch K., Moisoi N., Gandhi S., Kjaer S., Frith D., Harvey K., Deas E., Harvey R.J., McDonald N. (2007). The mitochondrial protease HtrA2 is regulated by Parkinson’s disease-associated kinase PINK1. Nat. Cell Biol..

[14] Tain L.S., Chowdhury R.B., Tao R.N., Plun-Favreau H., Moisoi N., Martins L.M., Downward J., Whitworth A.J., Tapon N. (2009). *Drosophila* HtrA2 is dispensable for apoptosis but acts downstream of PINK1 independently from Parkin. Cell Death Differ..

[15] Simon-Sanchez J., Singleton A.B. (2008). Sequencing analysis of *OMI/HTRA2* shows previously reported pathogenic mutations in neurologically normal controls. Hum. Mol. Genet..

[16] Ross O.A., Soto A.I., Vilarino-Guell C., Heckman M.G., Diehl N.N., Hulihan M.M., Aasly J.O., Sando S., Gibson J.M., Lynch T. (2008). Genetic variation of *Omi/HTRA2* and Parkinson’s disease. Parkinsonism Relat. Disord..

[17] Krüger R., Sharma M., Riess O., Gasser T., Van Broeckhoven C., Theuns J., Aasly J., Annesi G., Bentivoglio A.R., Brice A. (2011). A large-scale genetic association study to evaluate the contribution of *Omi/HTRA2* (PARK13) to Parkinson’s disease. Neurobiol. Aging.

[18] He Y-C., Huang P., Li Q-Q., Sun Q., Li D.H., Wang T., Shen J.Y., Du J.J., Cui S.S., Gao C. (2017). Mutation analysis of *HTRA2* gene in Chinese familial essential tremor and familial Parkinson’s disease. Parkinsons Dis..

[19] Yun J., Cao J.H., Dodson M.W., Clark I.E., Kapahi P., Chowdhury R.B., Guo M. (2008). Loss of-function analysis suggests that *Omi/HTRA2* is not an essential component of the *PINK1/PARKIN* pathway *in vivo*. J. Neurosci..

[20] Francione L.M., Annesley S.J., Carilla-Latorre S., Escalante R., Fisher P.R. (2011). The *Dictyostelium* model for mitochondrial disease. Semin. Cell Dev. Biol..

[21] Annesley S.J., Chen S., Francione L.M., Sanislav O., Chavan A.J., Farah C., De Piazza S.W., Storey C.L., Ilievska J., Fernando S.G. (2014). Dictyostelium, a microbial model for brain disease. Biochim. Biophys. Acta.

[22] Gaudet P., Pilcher K.E., Fey P., Chisholm R.L. (2007). Transformation of Dictyostelium discoideum with plasmid DNA. Nat. Protoc..

[23] Hoebeeck J., Speleman F., Vandesompele J. (2007). Real-time quantitative PCR as an alternative to Southern blot or fluorescence in situ hybridization for detection of gene copy number changes. Methods Mol. Biol..

[24] Annesley S.J., Bago R., Mehta A., Fisher P.R. (2011). A genetic interaction between NDPK and AMPK in *Dictyostelium discoideum* that affects motility, growth and development. Naunyn Schmied. Arch. Pharmacol..

[25] Eichinger L., Rivero F. (2006). Dictyostelium Discoideum Protocols.

[26] Annesley S.J., Carilla-Latorre S., Escalante R., Fisher P.R. (2013). Mitochondrial respiratory complex function and the phenotypic consequences of dysfunction. Methods Mol. Biol..

[27] Lin C-H., Chen M-L., Chen G.S., Tai C-H., Wu R-M. (2011). Novel variant Pro143Ala in *HTRA2* contributes to Parkinson’s disease by inducing hyperphosphorylation of HTRA2 protein in mitochondria. Hum. Genet..

[28] Chen S., Annesley S.J., Jasim R.A.F., Musco V.J., Sanislav O., Fisher P.R. (2017). The Parkinson’s disease-associated protein DJ-1 plays a positive nonmitochondrial role in endocytosis in *Dictyostelium* cells. Dis. Models Mech..

[29] Witke W., Nellen W., Noegel A. (1987). Homologous recombination in the *Dictyostelium* alpha-actinin gene leads to an altered mRNA and lack of the protein. EMBO J..

[30] Wilczynska Z., Barth C., Fisher P.R. (1997). Mitochondrial mutations impair signal transduction in *Dictyostelium discoideum* slugs. Biochem. Biophys. Res. Commun..

[31] Lay S.T., Sanislav O., Annesley S.J., Fisher P.R. (2016). Mitochondrial stress tests using Seahorse respirometry on intact *Dictyostelium discoideum* cells. Methods Mol. Biol..

[32] Gaudet P., Fey P., Basu S., Bushmanova Y.A., Dodson R., Sheppard K.A., Just E.M., Kibbe W.A., Chisholm R.L. (2011). Dictybase update 2011: Web 2.0 functionality and the initial steps towards a genome portal for the Amoebozoa. Nucl. Acids Res..

[33] Gray C.W., Ward R.V., Karran E., Turconi S., Rowles A., Viglienghi D., Southan C., Barton A., Fantom K.G., West A. (2000). Characterization of human HtrA2, a novel serine protease involved in the mammalian cellular stress response. Eur. J. Biochem..

[34] Sievers F., Wilm A., Dineen D.G., Gibson T.J., Karplus K., Li W., Lopez R., McWilliam H., Remmert M., Söding J. (2011). Fast, scalable generation of high-quality protein multiple sequence alignments using Clustal Omega. Mol. Syst. Biol..

[35] Claros M.G., Vincens P. (1996). Computational method to predict mitochondrially imported proteins and their targeting sequences. Eur. J. Biochem..

[36] Jones P., Binns D., Chang H-Y., Fraser M., Li W., McAnulla C., McWilliam H., Maslen J., Mitchell A., Nuka G. (2014). InterProScan 5: Genome-scale protein function classification. Bioinformatics.

[37] Suzuki Y., Imai Y., Nakayama H., Takahashi K., Takio K., Takahashi R. (2001). A serine protease, HtrA2, is released from the mitochondria and interacts with XIAP, inducing cell death. Mol. Cell.

[38] Small I., Peeters N., Legeai F., Lurin C. (2004). Predotar: A tool for rapidly screening proteomes for ***N***-terminal targeting sequences. Proteomics.

[39] Schiffer M., Edmundson A.B. (1967). Use of helical wheels to represent the structures of proteins and to identify segments with helical potential. Biophysical Journal.

[40] Barth C., Fraser D.J., Fisher P.R. (1998). Coinsertional replication is responsible for tandem multimer formation during plasmid integration into the *Dictyostelium* genome. Plasmid.

[41] Kotsifas M., Barth C., Lay S.T., de Lozanne A., Fisher P.R. (2002). Chaperonin 60 and mitochondrial disease in *Dictyostelium*. J. Muscle Res. Cell Motil..

[42] Bokko P.B., Francione L., Ahmed A.U., Bandala-Sanchez E., Annesley S.J., Huang X., Khurana T., Kimmel A.R., Fisher P.R. (2007). Diverse cytopathologies in mitochondrial disease are caused by AMP-activated protein kinase signaling. Mol. Biol. Cell.

[43] Carilla-Latorre S., Gallardo M.E., Annesley S.J., Calvo-Garrido J., Graña O., Accari S.L., Smith P.K., Valencia A., Garesse R., Fisher P.R. (2010). MidA is a putative mitochondrial methyltransferase required for mitochondrial complex I function. J. Cell Sci..

[44] Francione L.M., Fisher P.R. (2011). Heteroplasmic mitochondrial disease in *Dictyostelium discoideum*. Biochem. Pharmacol..

[45] Annesley S.J., Bandala-Sanchez E., Ahmed A.U., Fisher P.R. (2007). Filamin repeat segments required for photosensory signalling in *Dictyostelium discoideum*. BMC Cell Biol..

[46] Polgár L. (2005). The catalytic triad of serine peptidases. Cell Mol. Life Sci..

[47] Li W., Srinivasula S.M., Chai J., Li P., Wu J.W., Zhang Z., Alnemri E.S., Shi Y. (2002). Structural insights into the pro-apoptotic function of mitochondrial serine protease HtrA2/Omi. Nat. Struct. Biol..

[48] Martins L.M., Turk B.E., Cowling V., Borg A., Jarrell E.T., Cantley L.C., Downward J. (2003). Binding specificity and regulation of the serine protease and PDZ domains of HtrA2/Omi. J. Biol. Chem..

[49] Dagda R.K., Chu C.T. (2009). Mitochondrial quality control: Insights on how Parkinson’s disease related genes PINK1, parkin, and Omi/HtrA2 interact to maintain mitochondrial homeostasis. J. Bioenerg. Biomembr..

[50] Desideri E., Martins L.M. (2012). Mitochondrial stress signalling: HTRA2 and Parkinson’s disease. Int. J. Cell. Biol..

[51] Casadei N., Sood P., Ulrich T., Fallier-Becker P., Kieper N., Helling S., May C., Glaab E., Chen J., Nuber S. (2016). Mitochondrial defects and neurodegeneration in mice overexpressing wild-type or G399S mutant HtrA2. Hum. Mol. Genet..

