# Supplementary material for: Mitochondrial HTRA2 Plays a Positive, Protective Role in Dictyostelium discoideum but Is Cytotoxic When Overexpressed
Source: Genes (Basel). 2018 Jul 16;9(7):355. doi: 10.3390/genes9070355 (PMC6070809; doi:10.3390/genes9070355)
Supplement: Supplementary file 1 [file genes-09-00355-s001.pdf]

Supplemental Information

Supplementary text

1. Only half of the *D. discoideum* HTRA2 overexpression transformants contained the full length *htrA* gene in the overexpression construct (pPROF691)

To confirm the presence of the HTRA2 overexpression construct (pPROF691), PCR was performed to amplify *htrA* from pPROF691 using the primers A15PIF302 (the forward primer in A-15 promoter of PA15GFP vector backbone) and HOER (*htrA* reverse primer). Figure S3 shows a representative gel from the PCR assay. Only about half (26/50) transformants contained the full length *htrA* gene. Others contained deletions within the amplified fragment producing a smaller PCR product, while others produced no PCR product at all, despite being geneticin resistant (the selectable marker used in transformation). These results suggested that expression of HTRA2 at high levels might be lethal, so that only transformants with low copy numbers and/or mutations affecting the *htrA* gene could survive.

2. Copy numbers are low in half of the transformants containing the full length htrA gene in the overexpression construct (pPROF691)

To verify and further characterize transformants containing full length *htrA* genes in the overexpression construct, qPCR was performed to check the copy numbers of *htrA*. The results showed that 6 out of 12 HTRA2 overexpression transformants contained unusually low numbers of copies (<10) of the *htrA* amplicon (Table S2), not very different from the parental AX2 strain's single endogeneous copy. If HTRA2 overexpression is cytotoxic, these low copy number transformants may have been isolated successfully only because their ectopic expression levels of HTRA2 were sufficiently low to permit cell survival.

3. The recombinant *htrA* gene from higher copy number HTRA2 overexpression transformants is mutated

The preceding results showed that only about a quarter of the HTRA2 overexpression transformants contained the full length recombinant *htrA* in moderate to high copy numbers (>10). Since it had not been exposed to potentially mutagenic PCR reactions during cloning in *E. coli*, it was very unlikely that the A15-P promoter in the overexpression construct had suffered significant changes that could have affected the expression of HTRA2. Nevertheless, we sequenced this portion of pPROF691 from 298 bp upstream of the start codon and found that the A15-P promoter was intact. To determine if the *htrA* gene sequence itself was unaltered in the overexpression transformants, the sequences of the amplified *htrA* gene fragments from three *Dictyostelium* transformants (HPF1220, 1226 and 1227) with relatively high copy numbers (45, 63 and 253) were determined (multiple sequencing experiments from independently amplified PCR products, sequencing both strands). The results showed that the sequenced portion of the *htrA* gene in the construct within each tested transformant was mutated in the form of single base substitutions or insertions of extra base pair(s) (Fig. S4a), resulting in corresponding changes in the encoded protein (Fig. S4b).

Supplementary Figures.

VALUES OF COMPUTED PARAMETERS

Net charge of query sequence	: -13
Analysed region	: 82
Number of basic residues in targeting sequence	: 8
Number of acidic residues in targeting sequence	: 1
Cleavage site	: 49
Cleaved sequence	:

MIQSSIRKCLTSIKPNNIIGRSKVNIINFNINNRNIRYFASNSSSS

HYDROPHOBIC SCALE USED

		GES	KD	GvH1	ECS
H17	:	1.641	1.418	0.245	0.692
MesoH	:	-0.255	0.431	-0.262	0.269
MμHδ_075	:	31.167	24.707	9.070	8.466
MμHδ_095	:	31.396	22.015	8.106	8.185
MμHδ_100	:	32.492	19.315	7.730	7.975
MμHδ_105	:	37.183	20.126	10.934	8.269
Hmax_075	:	11.600	12.400	1.871	4.842
Hmax_095	:	7.613	15.225	-0.680	4.952
Hmax_100	:	5.600	9.400	-0.913	4.260
Hmax_105	:	8.750	14.117	3.373	4.888

PROBABILITYof export to mitochondria: 0.9447

Figure S1. Prediction of HTRA2's subcellular localization using MitoProt II. The full length HTRA2 protein (647 amino acids) was used to predict its probability of export to the mitochondria. The 82 N-terminal residues from the protein were analysed and a score of 0.9947 out of 1 was obtained, which predicts that HTRA2 is localized to the mitochondria. H17: the 17-residue segment of higher hydrophobicity in the sequence; MesoH: the average of the maximal hydrophobicity of a protein over an extended sequence length; MμHδ: the maximal Eisenberg's hydrophobic moment with 6 angles of *75"*, 95", 100" and 105", with a scanning window of 18 residues; Hmaxδ: the maximal hydrophobicity of each hydrophobic face in a helical structure; GES (Goldman, Engelman and Steitz scale): reflecting the circumstances in which amino acid residues appear in proteins by quantifying the free energy of water/oil transfer for residues in an α-helical structure; GvH1 (Gunnar von Heijne scale 1): a statistical scale obtained from the amino-acid-residue frequencies in the central part of a transmembrane segment with respect to the non-membranous stretches; KD (Kyte and Doolittle scale): considering values from water-to-vapor energy transfers and from internal/externa1 distribution of amino acid residues; ECS (Eisenberg's consensus scale), mitigating the effect of outlying values in any one scale, produced by the peculiarities of the method, and is a normalized average of four scales [1].

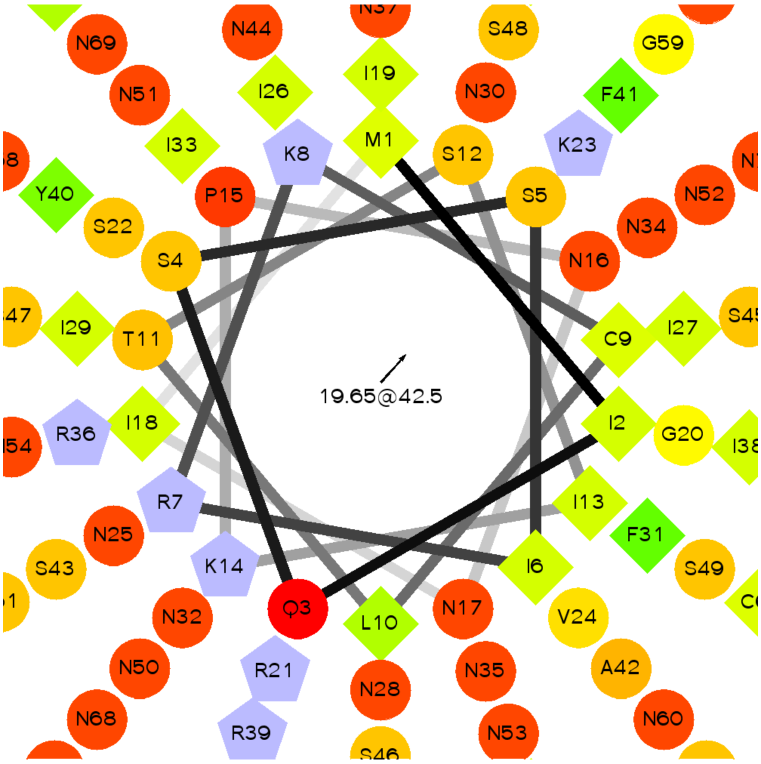

Figure S2: Prediction of HTRA2's subcellular localization using a Helical Wheel plot **[2].** The hydrophilic residues are shown as circles, hydrophobic residues as diamonds, potentially negatively charged residues as triangles, and potentially positively charged as pentagons. Hydrophobicity is color coded: the most hydrophobic residue is green, and the amount of green decreasing proportionally to the hydrophobicity, with zero hydrophobicity coded as yellow. Hydrophilic residues are coded red with pure red being the most hydrophilic (uncharged) residue, and the amount of red decreasing proportionally to the hydrophilicity. The potentially positively or negatively charged residues are light blue. The 18 amino acids from the N-terminus of HTRA2 contains 3 positively charged residues, 0 negatively charged residues, 8 hydrophilic and 7 hydrophobic residues. In addition, arginine (R7), is predicted to be recognized by mitochondrial processing peptidases and mitochondrial intermediate peptidases. These characteristics meet the requirements for import of HTRA2 to mitochondria [3].

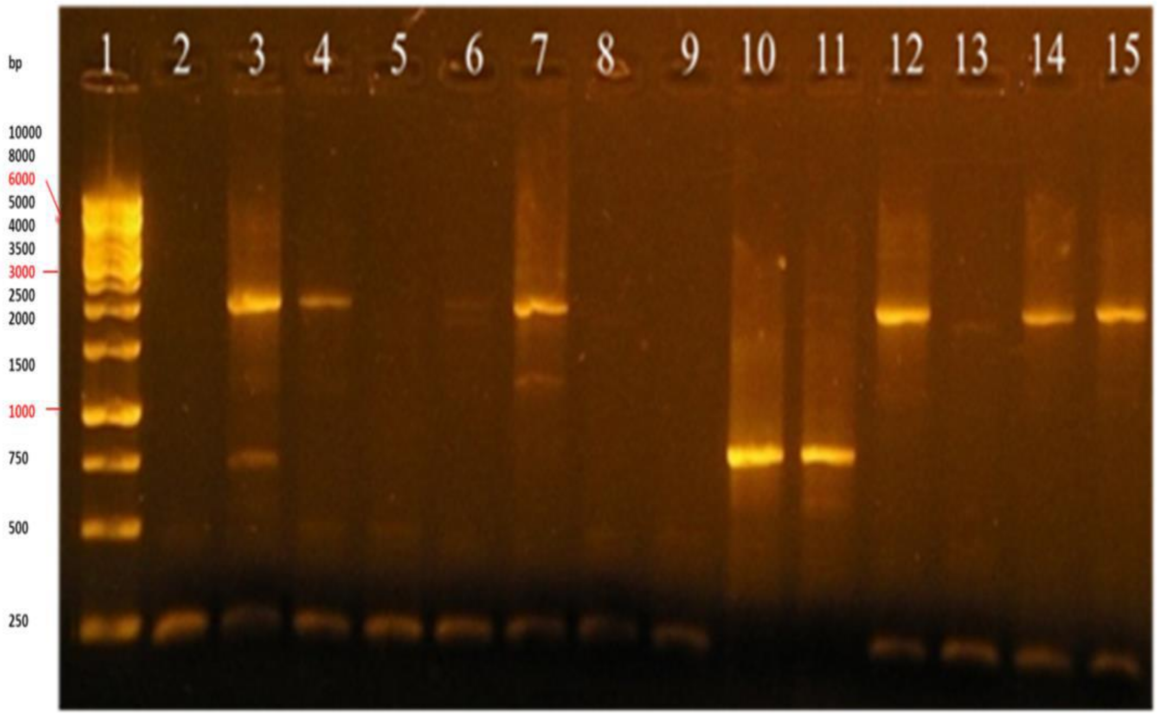

Figure S3: Amplification of *htrA* from HTRA2 overexpression transformants. The full length *htrA* gene is 2023 bp as seen in lanes 4, 7, 12, 13, 14 and 15. Lanes 10-11 contain a shorter fragment which may be a deleted form of *htrA*, whereas lanes 2, 5, 6, 8 and 9 don't contain any detectable *htrA* fragment. AX2 in lane 2 is used as the negative control as it does not contain the pA15GFP vector. The band of 250 bp in most of lanes (including untransformed AX2) may be due to nonspecific binding of the primers.

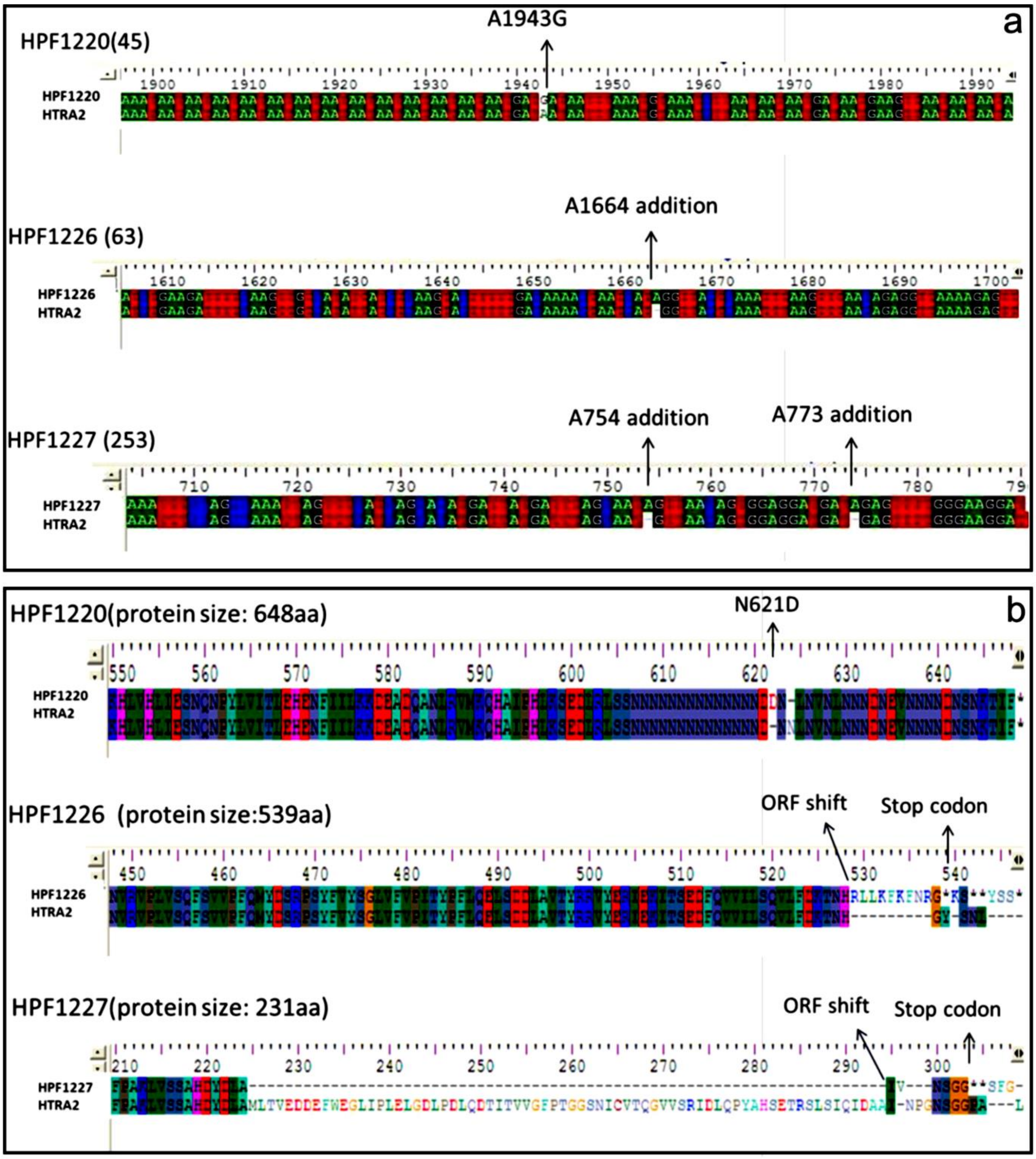

Figure S4: Partial sequence of HTRA2 from HTRA2 overexpression transformants**.** The gDNA from 3 HTRA2 overexpression transformants was extracted using DNAzol and PCR was performed to amplify the HTRA2 gene using A15PIF302 (the primer for the A15-P promoter) and HOER primers. The purified PCR amplicons were sequenced and the sequence was aligned with that of the native HTRA2 gene using Bioedit (a). The gene sequence was translated using on-line software at Expasy and aligned with the native HTRA2 protein sequence using Bioedit (shown in b). The mutations are indicated with arrows and the numbers are the mutation sites. These mutations resulted in an amino acid substitution (HPF1220) or Open Reading Frame (ORF) shifts (HPF1226, HPF1227) and downstream premature stop codon.

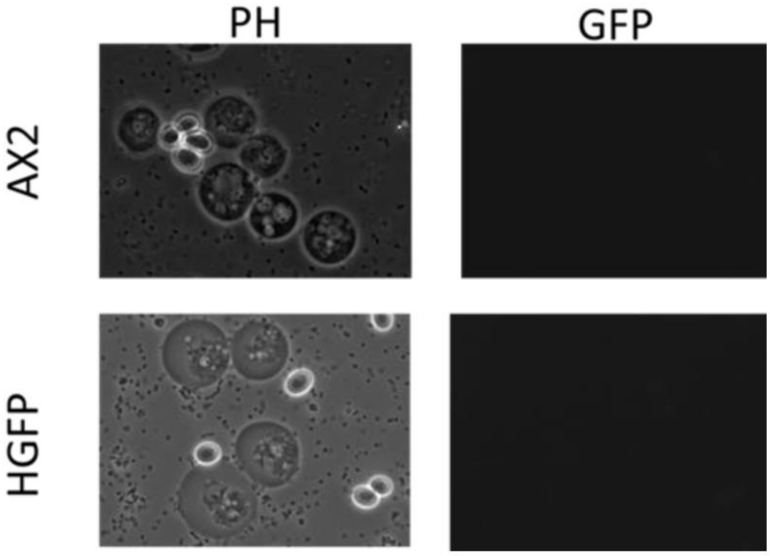

Figure S5: GFP fluorescence of HTRA2:GFP fusion (HGFP) transformants**.** PH: phase contrast GFP: green fluorescence protein – cells of a *D. discoideum* transformant containing the HTRA2:GFP fusion construct pPROF694. GFP fluorescence was not detected in the HTRA2:GFP transformants or in the wild type AX2.

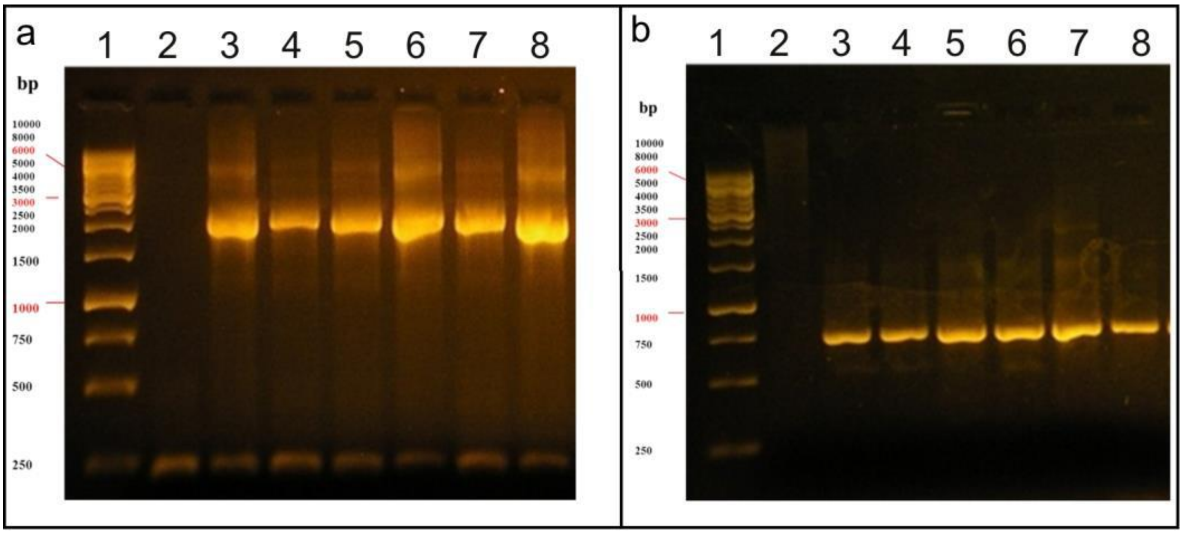

Figure S6: HTRA2:GFP fusion transformants with full length *htrA* and GFP sequences can be isolated. (**a)** Amplification of recombinant HTRA2 DNA from HTRA2:GFP transformants (lanes 3-8) and no amplification from AX2 (lane 2). The A15PIF302 and HOER primers were used to amplify a fragment containing the HTRA2 gene (2023 bp) and a portion of the A15-P promoter (302 bp). Together the two fragments constitute a 2325 bp amplicon. The band of approximately 250 bp in lanes 2-8 may be due to the nonspecific annealing of primers, particularly the primer for the AT-rich A15-P promoter. Lane 1 contains size markers. **(b)** Amplification of GFP DNA (750 bp) from HTRA2:GFP transformants (lanes 3-8) and no amplification from AX2 (lane 2). Lane 1 contains size markers.

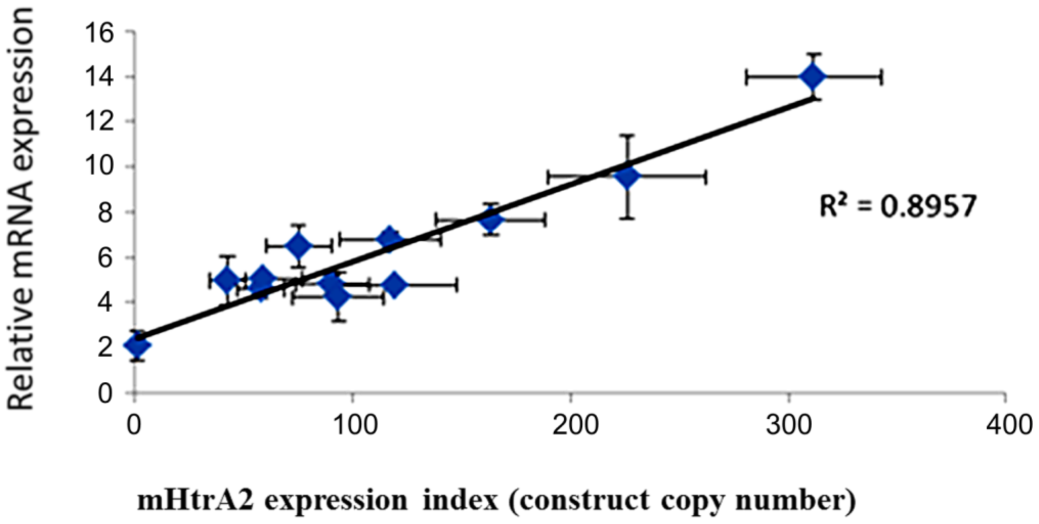

Figure S7: Expression of $HTRA2_{S300A}$ mRNA correlates with the number of copies of mutated *htrA*. The copy numbers of pPROF692 encoding $HTRA2_{S300A}$ were determined by qPCR and the relative $HTRA2_{S300A}$ mRNA levels were measured by qRT-PCR and normalized against the reference gene filamin. $HTRA2_{S300A}$ mRNA expression levels positively correlate with the copy numbers of pPROF692 ($p = 1.57 \times 10^{-6}$, F test, n = 12 independent transformants). Error bars are standard errors from 3 independent experiments, each involving duplicate measurements.

Supplementary Tables.

Table S1: Prediction of HTRA2's subcellular localization using Predotar [4].

Gene	Mitochondrial	ER	Elsewhere	Prediction
HTRA2	0.56*	0.01	0.43	mitochondria

*The most probable localization of HTRA2 is to the mitochondria.

Table S2: Copy numbers of *htrA* in HTRA2 overexpression transformants

Strains	Copy numbers
AX2	1
HPF1220	45
HPF1221	31
HPF1222	5
HPF1223	2
HPF1224	1
HPF1225	2
HPF1226	63
HPF1227	253
HPF1228	9
HPF1229	6
HPF1230	45
HPF1231	21

References

1. M.G. Claros, P. Vincens. Computational method to predict mitochondrially imported proteins and their targeting sequences. *Eur. J. Biochem.* **1996**, *241*, 779-786.
2. Schiffer, M.; Edmundson, A.B. Use of helical wheels to represent the structures of proteins and to identify segments with helical potential. *Biophysical Journal* **1967**, *7*, 121–35.
3. Endo, T; Kohda, D. Functions of outer membrane receptors in mitochondrial protein import. *Biochim Biophys Acta.* **2002**, *1592*, 3-14.
4. Small, I.; Peeters, N.; Legeai, F.; Lurin, C. Predotar: A tool for rapidly screening proteomes for *N*-terminal targeting sequences. *Proteomics* **2004**, *4*, 1581-1590.
